# Greater mesophyll conductance and leaf photosynthesis in the field through modified cell wall porosity and thickness via AtCGR3 expression in tobacco

**DOI:** 10.1111/pbi.14364

**Published:** 2024-04-30

**Authors:** Coralie E. Salesse‐Smith, Edward B. Lochocki, Lynn Doran, Benjamin E. Haas, Samantha S. Stutz, Stephen P. Long

**Affiliations:** ^1^ Carl R. Woese Institute for Genomic Biology University of Illinois at Urbana‐Champaign Urbana IL USA; ^2^ Departments of Plant Biology and of Crop Sciences University of Illinois at Urbana‐Champaign Urbana IL USA

**Keywords:** mesophyll conductance, CO_2_ assimilation, carbon isotope discrimination, *AtCGR3* pectin methyltransferase, water use efficiency, photosynthetic efficiency

## Abstract

Mesophyll conductance (*g*_m_) describes the ease with which CO_2_ passes from the sub‐stomatal cavities of the leaf to the primary carboxylase of photosynthesis, Rubisco. Increasing *g*_m_ is suggested as a means to engineer increases in photosynthesis by increasing [CO_2_] at Rubisco, inhibiting oxygenation and accelerating carboxylation. Here, tobacco was transgenically up‐regulated with *Arabidopsis* Cotton Golgi‐related 3 (*CGR3*), a gene controlling methylesterification of pectin, as a strategy to increase CO_2_ diffusion across the cell wall and thereby increase *g*_m_. Across three independent events in tobacco strongly expressing *AtCGR3*, mesophyll cell wall thickness was decreased by 7%–13%, wall porosity increased by 75% and *g*_m_ measured by carbon isotope discrimination increased by 28%. Importantly, field‐grown plants showed an average 8% increase in leaf photosynthetic CO_2_ uptake. Up‐regulating *CGR3* provides a new strategy for increasing *g*_m_ in dicotyledonous crops, leading to higher CO_2_ assimilation and a potential means to sustainable crop yield improvement.

## Introduction

Photosynthesis, the process of converting light energy and atmospheric CO_2_ into organic compounds, is directly or indirectly the source of all food. Improving photosynthetic efficiency has become a major research objective in order to feed an increasing global population, and to supplement ongoing crop breeding efforts (Bailey‐Serres *et al*., 2019; Ray *et al*., 2013). A critical need is to achieve increases without the use of more land or water, given pressures on supply and diminished soil moisture under climate change (Dai, 2013; Hunter *et al*., 2017; Ort and Long, 2014). One strategy with the potential to help meet this challenge is to use genetic engineering to increase photosynthetic efficiency of C_3_ plants via increased mesophyll conductance (Flexas *et al*., 2013; Leakey *et al*., 2019; Lundgren and Fleming, 2020). However, in order to test this, there is a need to gain a better understanding of mesophyll conductance and how manipulating it may affect photosynthesis and water use efficiency.

Mesophyll conductance (*g*_m_) is a measure for the ease with which CO_2_ from the sub‐stomatal cavities may diffuse to the chloroplast stroma, where it is fixed by Rubisco. Increasing *g*_m_ can increase photosynthetic capacity of C_3_ plants, and potentially crop yields, by increasing the concentration of CO_2_ around Rubisco (Flexas *et al*., 2013; Perez‐Martin *et al*., 2009). This would decrease photorespiratory losses and accelerate carboxylation, without any additional cost in transpiration (Flexas *et al*., 2013; Leakey *et al*., 2019). A combination of factors are considered to affect *g*_m_. These include gas phase diffusion from the inside of the stomata to exposed mesophyll cell walls and then liquid phase diffusion through the cell wall, plasma membrane, cytosol, chloroplast envelope and chloroplast stroma (Cousins *et al*., 2020; Evans, 2021; Salesse‐Smith *et al*., 2023; Xiao and Zhu, 2017).

Mesophyll conductance is influenced by several leaf anatomical properties (Evans, 2021). Among these are the chloroplast surface area exposed to intercellular airspaces (*S*_c_), the mesophyll surface area exposed to intercellular airspaces (*S*_m_) and their ratio (*S*_c_/*S*_m_), the latter of which has been shown to be positively correlated with *g*_m_ (Ren *et al*., 2019). Mesophyll cell wall thickness (*T*_cw_) and porosity, as well as the permeability of the plasma membrane and chloroplast envelope to CO_2_, are also considered important properties affecting *g*_m_ (Carriquí *et al*., 2020; Flexas *et al*., 2021). Both aquaporins and plastid surface area have been suggested to affect *g*_m_ (Lundgren and Fleming, 2020; Momayyezi *et al*., 2020). However, manipulation studies have produced mixed results (Głowacka *et al*., 2023; Heckwolf *et al*., 2011; Kromdijk *et al*., 2020; Uehlein *et al*., 2008). Several modelling studies have suggested that the cell wall is one of the most prominent constraints on *g*_m_ (Gago *et al*., 2020; Xiao and Zhu, 2017; Yin and Struik, 2017). Cell wall conductance to CO_2_ (*g*_cw_) depends on its thickness, the tortuosity of the path of CO_2_ through the pores of the cell wall (*τ*), and the number of those pores (porosity *p*) (Evans, 2021). Previous studies, including one on natural variation with leaf age in tobacco leaves, have reported that 1/*g*_m_ has a strong positive correlation with cell wall thickness, inferring that decreasing cell wall thickness is a means to increase *g*_m_ (Clarke *et al*., 2021; Onoda *et al*., 2017).

Cell wall formation is a complex process involving many genes and their protein products, so there are many potential options for altering cell wall thickness. Previous studies in *A. thaliana* have shown that overexpression of Cotton Golgi‐related 3 (*AtCGR3*) or a functionally redundant gene *AtCGR2* increased the fraction of intercellular airspaces (*f*_ias_) and plant growth (Kim *et al*., 2015; Weraduwage *et al*., 2016). CGR3 is a pectin methyltransferase that catalyses the methylesterification of pectin in the cell wall (Kim *et al*., 2015). Essentially, CGR3 adds methyl groups to pectin, serving to increase the extensibility of the cell wall, while affecting porosity (Weraduwage *et al*., 2016; Wu *et al*., 2018). Pectin is one of the three main components of dicot primary cell walls, along with cellulose and hemicellulose. Increasing the ratio of pectin to cellulose and hemicellulose or increasing pectin methylation may result in increased cell wall porosity (Flexas *et al*., 2021; Roig‐Oliver *et al*., 2021). However, neither cell wall thickness, porosity or mesophyll conductance were measured in these prior studies overexpressing CGR3 or CGR2 (Kim *et al*., 2015; Weraduwage *et al*., 2016).

We hypothesized that genetically up‐regulating CGR3 may improve CO_2_ diffusion through the cell wall by decreasing its thickness and increasing its porosity, and further hypothesized this, in turn, would increase *g*_m_, CO_2_ concentration at Rubisco (*C*_c_) and, leaf CO_2_ uptake rate (*A*). This was tested by engineering *AtCGR3* into tobacco and molecular and physiological phenotyping of the resulting events in controlled environments and in the field as a test of concept.

## Results

### Transgenic tobacco expressing AtCGR3

**Figure 1:**
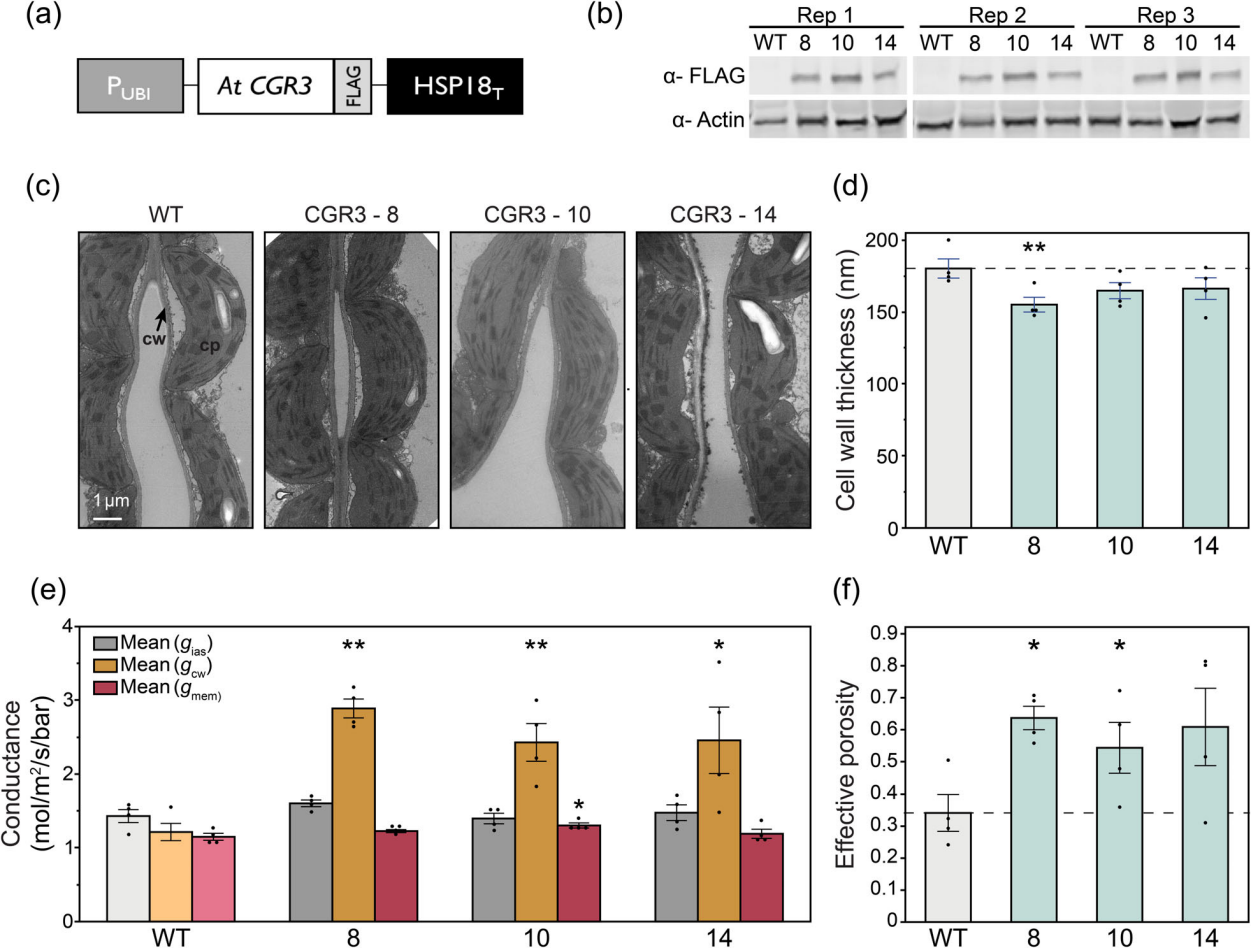
AtCGR3 protein expression in tobacco and its effect on CO_2_ conductance across the cell wall. (a) Transgene designed to constitutively express an *Arabidopsis* pectin methyltransferase CGR3. The transgene was stably transformed into tobacco cv. Samsun. (b) Total soluble protein isolated on a leaf area basis from single copy T2 homozygous plants and analysed by immunoblot. Three transgenic events (8, 10 and 14) and the wild‐type (WT) control were probed with anti‐FLAG and anti‐Actin antibodies. CGR3 protein is ~28 kDa. Actin was used as a loading control. (c) Representative transmission electron micrographs for each event. cw, cell wall; cp, chloroplast. (d) Mesophyll cell wall thickness was measured from electron micrographs. (e) Estimated CO_2_ conductance across the intercellular airspace (*g*_ias_), cell wall (*g*_cw_) and membranes (*g*_mem_), expressed on a leaf area basis. (f) Estimated effective porosity (*p*/*τ*) of the cell wall. Values are shown as the mean ± SEM (*n* = 4). Asterisks indicate significant differences between WT and the CGR3 transgenic line (**P < 0.05, *P < 0.1); one‐way ANOVA, Dunnett's *post hoc* test; *g*_cw_ significance determined with Welch ANOVA, Games‐Howell *post hoc* test.

A construct expressing the *Arabidopsis* pectin methyltransferase CGR3 was designed to test the hypothesis that up‐regulating CGR3 will decrease the thickness and increase the porosity of the cell wall to improve mesophyll conductance. This construct contains the *Arabidopsis* ubiquitin 10 promoter and 5′ leader to drive constitutive expression of *AtCGR3*. As antibodies were not available, a C‐terminal FLAG epitope tag was included before the *Arabidopsis* heat shock protein 18 terminator (Figure 1a). The construct was stably transformed into tobacco cv. Samsun, and T2 homozygous plants from three independent single insertion events were characterized in the greenhouse and field. Non‐transgenic wildtype (WT) tobacco plants of the genotype transformed and equivalent generation propagated in the same environment were used as controls.

qPCR analysis confirmed that all transgenic lines had high levels of *AtCGR3* RNA expression, while no expression was detected in the WT controls (Figure S1). Immunoblotting was then used to ensure that *At*CGR3 protein was accumulating in the transgenic tobacco plants. Strong CGR3 protein expression was observed exclusively in the transgenic plants when probed with anti‐FLAG (Figure 1b).

### AtCGR3 expression increases CO_2_ conductance across the cell wall

Mesophyll chloroplast ultrastructure observed by transmission electron microscopy showed no differences between genotypes (Figure 1c). Mesophyll cell wall thickness (*T*_cw_) was decreased by 7%–13% in the transgenic plants expressing *AtCGR3* (Figure 1d).

**Figure 2:**
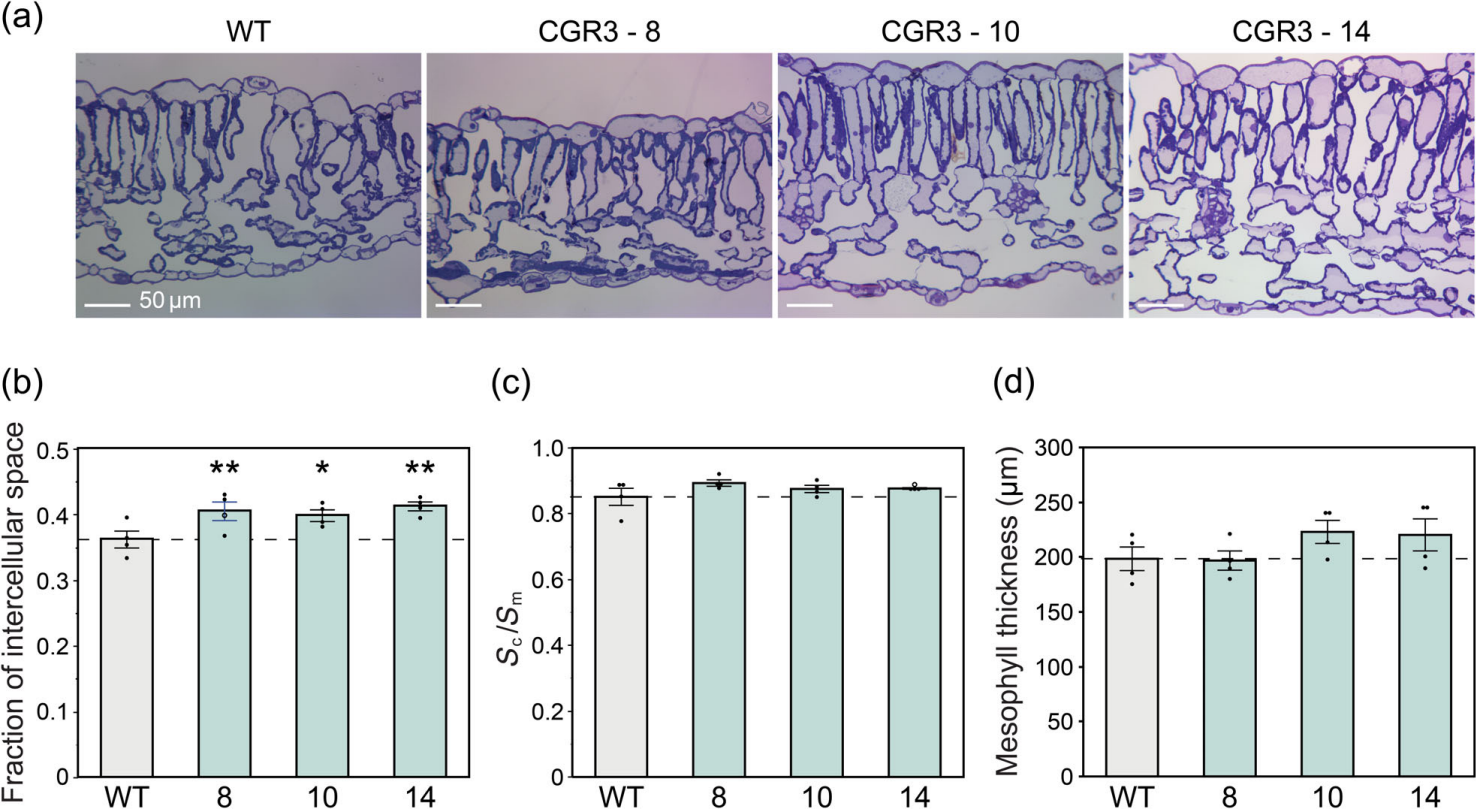
Light micrographs of transverse leaf sections and measured leaf anatomical traits. (a) Representative light micrographs. Light micrographs were used to measure (b) fraction of intercellular airspace (c) ratio of chloroplast surface area exposed to intercellular airspaces (*S*_*c*_) to mesophyll surface area exposed to intercellular airspaces (*S*_*m*_), and (d) mesophyll thickness. Values are shown as the mean ± SEM (*n* = 4 plants). Asterisks show significant differences between WT and the CGR3 transgenic line (**P < 0.05, *P < 0.1); (b) and (d) one‐way ANOVA, Dunnett's *post hoc* test; (c) Wilcoxon's non‐parametric test.

**Figure 3:**
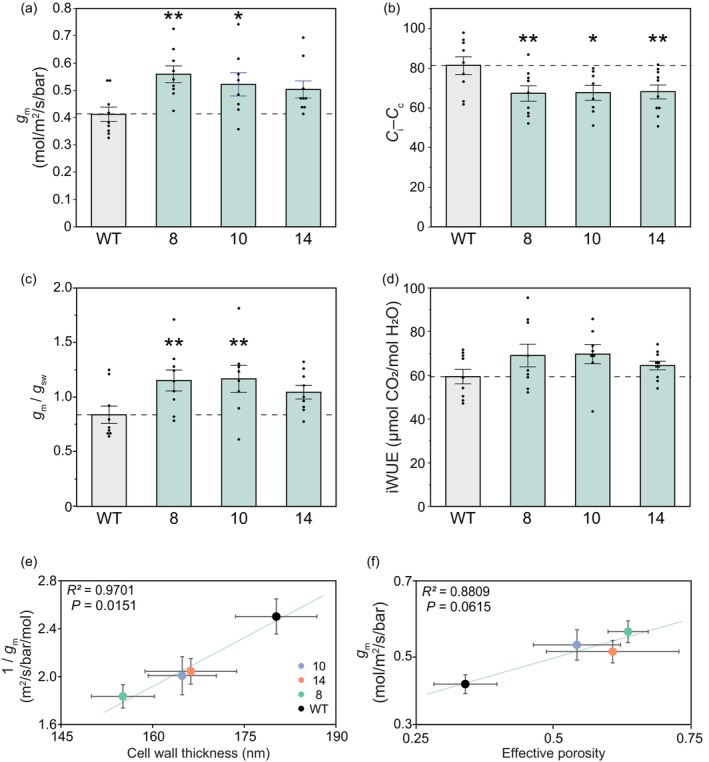
Mesophyll conductance and associated parameters estimated from carbon isotope discrimination (Δ^13^C) coupled with gas exchange at 2% oxygen in greenhouse‐grown tobacco. (a) Mesophyll conductance (*g*_m_) calculated from Δ^13^C, (b) the drawdown of CO_2_ into the chloroplast (*C*_*i*_–*C*_*c*_), (c) the ratio of mesophyll conductance (*g*_m_) to stomatal conductance (*g*_sw_) and (d) intrinsic water use efficiency (iWUE), i.e. the ratio of net CO_2_ assimilation rates (*A*) to stomatal conductance (*g*_sw_). Measurements were made under the following conditions: light intensity of 1800 μmol m^‐2^ s^‐1^, leaf temperature of 25 °C, 2% O_2_ and 400 μmol mol^‐1^ CO_2_. Asterisks indicate significant differences between WT and the CGR3 transgenic line (**P < 0.05, *P < 0.1); one‐way ANOVA, Dunnett's post hoc test. (e) The relationship between 1/*g*_m_ and mesophyll cell wall thickness and, (f) the relationship between *g*_m_ and effective porosity. The solid lines represent linear regressions from the data points calculated using Pearson's coefficient of correlation. Values are shown as the mean ± SEM (*n* = 4).

*g*_m_ includes CO_2_ diffusion across multiple sequential barriers, each of which has an associated conductance *g*. The conductance across the intercellular airspace (*g*_ias_), cell wall (*g*_cw_) and membranes (*g*_mem_) are expected to have the largest effects on *g*_m_ and can be estimated using measured values of *g*_*m*_, *f*_ias_, *T*_cw_, *T*_mes_ and *S*_*c*_ (Evans and von Caemmerer, 2013; Xiong, 2023). Using the corresponding measured values presented in Figures 1, 2, 3, we calculated that plants expressing AtCGR3 had a significantly increased *g*_cw_ of 114%, with no significant changes in *g*_ias_ or *g*_mem_ (Figure 1e). *g*_cw_ is directly influenced by cell wall thickness (Figure 1d), porosity and tortuosity (Flexas *et al*., 2021). Effective porosity (*p*/*τ*) of the cell wall was calculated to have increased by an average of 75% compared to the WT control (Figure 1f).

Representative light micrograph images (Figure 2a) show differences in leaf and mesophyll thickness (*T*_mes_). CGR3 expression resulted in significant increases in the fraction of intercellular airspace (*f*_ias_) of approximately 12% (Figure 2b), as well as minor increases in *S*_c_/*S*_m_ in the three independent transgenic events (Figure 2c). *T*_mes_ was increased in two of the three independent transgenic events (Figure 2d). Small decreases in leaf mass per unit area (LMA) were observed; however, these differences were not significantly different from WT (Table S2).

To explore whether CGR3 expression altered cell wall composition, we measured cell wall pectin, hemicellulose and cellulose content. No primary cell wall component showed any significant differences between CGR3 and WT (Table S2). Additionally, there was no difference in the ratio of pectin content to the sum of hemicellulose and cellulose content, a value used to indicate cell wall porosity (Table S2) (Flexas *et al*., 2021).

### Increases in *g*_m_ estimated from Δ ^13^C in transgenic lines grown under controlled growth conditions

Mesophyll conductance was measured to assess whether the anatomical changes described above, including decreased *T*_cw_ and increased *f*_ias_, affect CO_2_ diffusion. Multiple methods were used to overcome some of the uncertainties associated with estimating *g*_m_. First, carbon isotope discrimination (Δ^13^C) measurements coupled with gas exchange at 2% oxygen were used to estimate *g*_m_ in greenhouse‐grown tobacco. Δ^13^C measurements showed that *g*_m_ was increased in all three events by an average of 28% relative to WT (Figure 3a). Concomitantly, all three events showed a significantly smaller drawdown of [CO_2_] between the stomatal cavity and chloroplast stroma (*C*_i_ – *C*_c_), averaging a 20% smaller drawdown than WT and, therefore, a greater [CO_2_] at Rubisco (Figure 3b). No changes in stomatal conductance (*g*_sw_) were observed, resulting in significant increases in the ratio of *g*_m_/*g*_sw_ (Figure 3c). Small increases in CO_2_ assimilation (*A*) were observed, resulting in indicated increases in intrinsic water use (iWUE; Figure 3d) in all three events, although these were not statistically significant.

Total leaf sugar and starch trended higher in all three transgenic events relative to WT, consistent with increased CO_2_ assimilation (Figure S2). To check for pleiotropic effects from increasing *g*_m_, stomatal density and chlorophyll content were measured. All genotypes had similar adaxial and abaxial stomatal densities (Figure S3a,b) and no change in the ratio of abaxial:adaxial stomatal densities was observed (Figure S3c). In addition, leaf chlorophyll content, as measured using a SPAD meter, did not differ between WT and transgenic plants (Table S2).

In addition, 1/*g*_m_ was significantly lower in all CGR3 events (Table S2) and showed a positive correlation with cell wall thickness (*P* = 0.0151, *R* ^2^ = 0.97, Figure 3e), consistent with previous studies (Clarke *et al*., 2021; Ren *et al*., 2019). In addition, *g*_m_ was significantly correlated with effective porosity (*P* = 0.0615, *R* ^2^ = 0.88, Figure 3f).

### Increased *g*_m_ in AtCGR3 events confirmed under field conditions using the variable *J* method

Subsequently, a field experiment was conducted to assess whether differences observed in *g*_m_ under greenhouse conditions were reproduced under field conditions. In 2022, a field experiment was carried out with replicated plots of the same three independent transgenic events overexpressing AtCGR3, using a randomized block design (Figure S5a).

**Figure 4:**
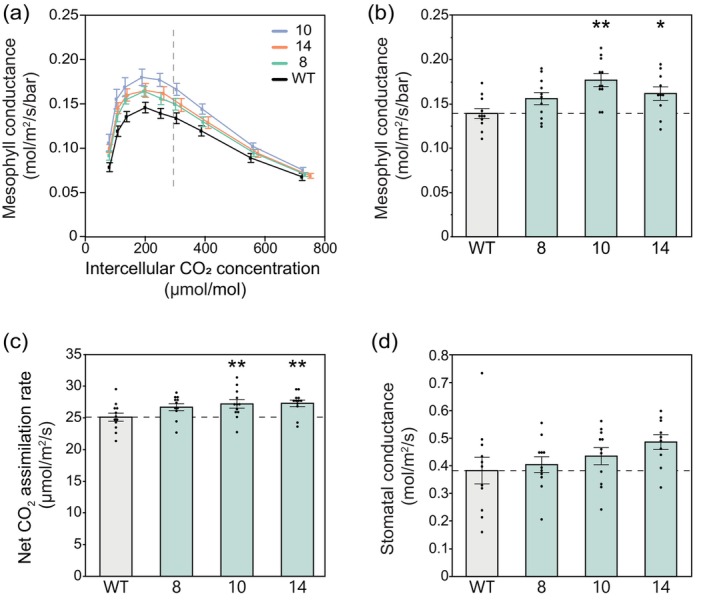
CO_2_ assimilation measured with gas exchange in parallel with chlorophyll fluorescence to estimate mesophyll conductance in field‐grown tobacco plants. (a) Mesophyll conductance (*g*_*m*_) as a function of intercellular CO_2_ concentration, estimated using the variable *J* method. The vertical dashed line shows the average operating *C*_*i*_ (where ambient CO_2_ is 420 μmol mol^‐1^). (b) Mesophyll conductance measured at 400 μmol mol^‐1^ CO_2_ derived from (a). (c) Net CO_2_ assimilation rates and (d) stomatal conductance to water (*g*_sw_), each measured at 400 μmol mol^‐1^ CO_2_. (a–d) measurements made at light intensity of 1800 μmol m^‐2^ s^‐1^, leaf temperature of 28 °C and 60% humidity. Values are shown as the mean ± SEM (*n* = 10–11). Asterisks indicate significant differences between WT and the CGR3 transgenic line (**P < 0.05, *P < 0.1); one‐way ANOVA, Dunnett's *post hoc* test.

Gas exchange measurements were made on the field‐grown plots to evaluate the physiological effects of decreasing thickness and increasing porosity of the mesophyll cell walls. To test if *g*_m_ was altered, gas exchange measurements were made in parallel with chlorophyll fluorescence measurements. We measured CO_2_ assimilation rates (*A*) as a function of intercellular CO_2_ concentrations (*C*_i_) under saturating light and fit the *A*–*C*_i_ curves using the variable *J* method to derive *g*_m_ (Harley *et al*., 1992; Moualeu‐Ngangue *et al*., 2017). This method models the relationship between *A*, the electron transport rate (*J*), and *C*_c_ to estimate *g*_m_ over a range of intercellular [CO_2_] (Figure 4a) and was used because the tunable laser diode system is not field portable. For each genotype, g_m_ exhibits a maximum for *C*_*i*_ just below the operating point and decreases for significantly larger or smaller *C*_*i*_, in agreement with previous measurements (Busch *et al*., 2020; Flexas *et al*., 2007). Measurements made at approximately ambient [CO_2_] (400 μmol mol^‐1^ CO_2_) showed an average 18% increase in *g*_m_ in the CGR3 transgenic plants that were statistically significant, consistent with the prior greenhouse study (Figure 4b). Importantly, and consistent with increased [CO_2_] at Rubisco, CO_2_ assimilation rates were significantly increased by an average of 8% in the CGR3 plants relative to the WT controls (Figure 4c). However, *g*_sw_ was also marginally increased (Figure 4d), resulting in no significant change in intrinsic water use efficiency in the field. No change in the slope of *A* versus *g*_sw_ was apparent between WT and the transgenic plants (Figure S6a). Both *g*_m_ and *g*_sw_ had a strong positive correlation with CO_2_ assimilation (Figure S6).

### *V*_*c*,max_ and apparent *V*_*c*,max_ estimates from field gas exchange measurements consistent with increased *g*_m_ in AtCGR3 transgenic plants

**Figure 5:**
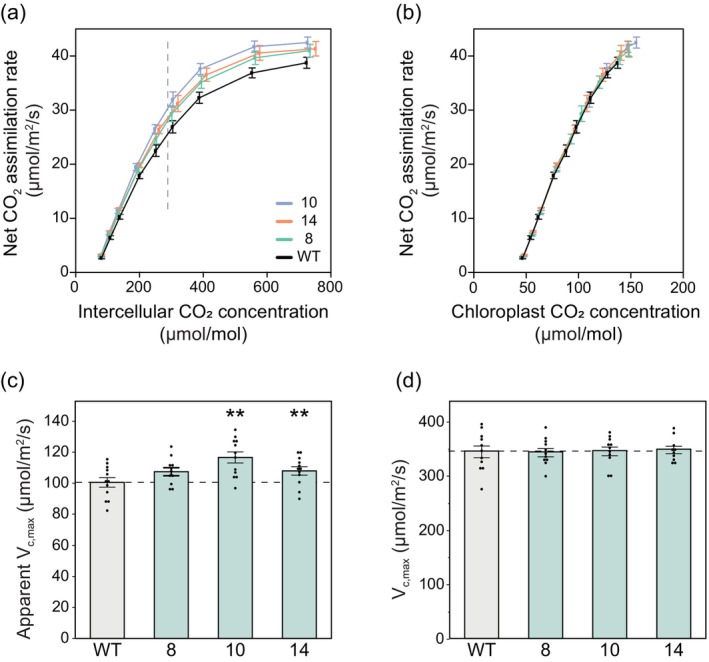
CO_2_ response curves and maximum rates of Rubisco carboxylation based on intercellular [CO_2_] and chloroplast [CO_2_]. (a) Response of net CO_2_ assimilation to intercellular [CO_2_] (*C*_*i*_). Measurements were made under the following conditions: light intensity of 1800 μmol m^‐2^ s^‐1^, leaf temperature of 28 °C and 60% humidity. CO_2_ concentrations varied from 20 to 1800 μmol mol^‐1^ CO_2_. The vertical dashed line is the average operating *C*_*i*_ (where ambient CO_2_ is 420 μmol mol^‐1^). (b) Response of net CO_2_ assimilation to chloroplast [CO_2_] (*C*_*c*_). *C*_*c*_ estimated from variable *J* fits. (c) Apparent maximum Rubisco carboxylation rate (*V*_*c*,max_) values at 25 °C estimated from response curves in panel a. *g*_m_ equal to infinity. (d) Maximum Rubisco carboxylation rate (*V*_*c*,max_) values at 25 °C estimated from response curves in panel b. *g*_*m*_ equal to estimated values from variable *J* method (Figure 4a). Values are shown as the mean ± SEM (*n* = 10–11). Asterisks indicate significant differences between WT and the CGR3 transgenic line (**P < 0.05); one‐way ANOVA, Dunnett's *post hoc* test.

The measured *A*–*C*_i_ responses (Figure 5a) were fit to the Farquhar–von Caemmerer–Berry model (von Caemmerer, 2000) to estimate the apparent maximum rate of Rubisco carboxylation (*V*_*c*,max_) (Figure 5c). The apparent *V*_*c*,max_ value is determined by the initial phase of the relationship of *A* to intercellular [CO_2_] (*C*_i_), so it is a function of both the actual activity of Rubisco and *g*_m_. To test whether the increase in apparent *V*_*c*,max_ in the transgenic events was the result of increased *g*_m_ or a pleiotropic effect on Rubisco activity, the curves were re‐analysed on a *C*_c_ basis (Figure 5b), whose values were derived from the *g*_m_ obtained at each [CO_2_] (Figure 4a). The initial phase (*V*_*c*,max_) of the transgenic *A*–*C*_*c*_ curves overlies that of the WT (Figure 5b), inferring that the difference was entirely due to increased *g*_m_ and not Rubisco activity (Figure 5d).

### Biomass maintained in field‐grown plants with decreased cell wall thickness and increased porosity

**Figure 6:**
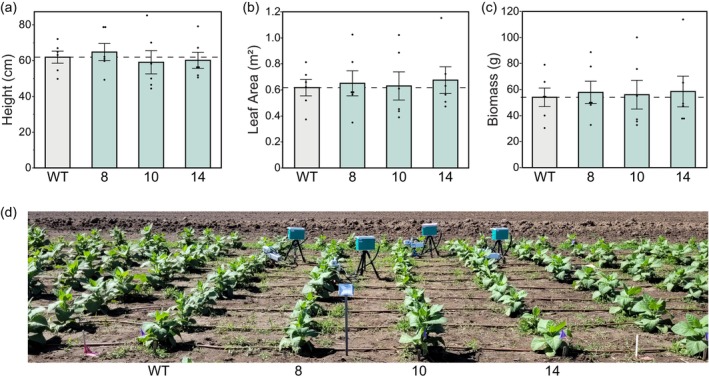
Plant growth traits in field‐grown tobacco plants. (a) Plant height, (b) leaf area and (c) biomass (sum of leaf, stem and root dry weights). Values are shown as the mean ± SEM (*n* = 6 plots). Asterisks indicate significant difference between WT and the CGR3 transgenic line (**P < 0.05, *P < 0.1); (a) and (b) one‐way ANOVA, Dunnett's *post hoc* test; (c) Wilcoxon's non‐parametric test. (d) Tobacco plants growing in the field in Urbana, Illinois summer 2022.

Cell walls function to protect plants from biotic and abiotic stresses as well as provide structural integrity to the plant, facilitate normal growth and play a crucial role in water relations (Hückelhoven, 2007; Taiz, 1984). To assess whether decreased *T*_cw_ had any negative impacts on plant growth and form in the field, we measured a number of plant growth traits (Figure 6). No significant differences in plant height, leaf area or total dry biomass were observed between the transgenic lines and control plants (Figure 6a–c). In addition, there were no changes in leaf number or biomass of leaves, stems or roots when weighed individually (Table S3). We did not observe any differences in structural integrity, lodging, pest or pathogen stress between the AtCGR3 and WT plants; the transgenic lines were essentially indistinguishable from the WT controls (Figure 6d). These results are consistent with growth measurements made in the greenhouse, with the exception that leaf number was significantly increased in the AtCGR3 lines in the greenhouse (Figure S4).

## Discussion

Increasing the diffusive conductance of CO_2_ from the atmosphere to Rubisco has been frequently proposed as an important target for improving CO_2_ assimilation in C_3_ species (Flexas *et al*., 2013, 2016; Lundgren and Fleming, 2020; Zhu *et al*., 2010). Yet, there have been few successes in engineering a change in *g*_m_ into crops. This is at least partly due to an incomplete mechanistic understanding of *g*_m_. While aquaporin channels in the plasma membrane and chloroplast surface area were considered prime targets, manipulations have had no or mixed success for C_3_ species (Głowacka *et al*., 2023; Kromdijk *et al*., 2020). However, observations of variation in both thickness and porosity of the cell wall indicated these as another means to increase mesophyll conductance (Clarke *et al*., 2021; Flexas *et al*., 2021). We identified overexpression of *AtCGR3* as an opportunity to both increase porosity and decrease thickness of the cell wall. Three independent overexpression events in tobacco showed, on average, a 75% increase in porosity and a 10% decrease in thickness of the mesophyll cell walls. This corresponded to a 28% increase in *g*_m_ (estimated using two independent methods) and an 8% increase in leaf CO_2_ assimilation rates, without any pleiotropic effects. This study provides the first report of increased mesophyll conductance via increased porosity and decreased thickness of the cell wall in a dicot species. It also appears one of few demonstrated transgenic increases in mesophyll conductance and leaf photosynthesis of a crop within a replicated field trial (Xu *et al*., 2019). This should serve as a proven test‐of‐concept for further manipulations of the cell wall and application to food crops.

Measured values of *g*_m_ are subject to uncertainty because the trait cannot be determined directly and must be estimated using indirect methods. Thus, it is important to check for consistency across different techniques (Pons *et al*., 2009) and growth environments. Here, although absolute values are different, similar relative increases in *g*_m_ were observed in each of the three transgenic events relative to WT, both when estimated in the field from chlorophyll fluorescence and in the greenhouse from isotopic^13^C measurements (Figures 3, 4). Previous studies have also shown that isotopic^13^C measurements result in higher estimates of *g*_m_ than the fluorescence variable *J* method (Kromdijk *et al*., 2020; Xiong, 2023). A recent study comparing *g*_m_ values measured using both carbon isotope discrimination and chlorophyll fluorescence showed that isotopic measurements were consistently higher than fluorescence measurements, with up to a threefold difference estimated from measurements made on the same leaf (Xiong, 2023). Reasons for this remain unclear and require further investigation. However, the two sets of values showed a strong linear correlation, indicating that comparisons within each method should be valid. The low *g*_m_ values estimated using fluorescence also lead to an overestimation of absolute *V*_c,max_ values; however, this has no effect on relative differences between genotypes. Associated measurements of *A* provide another consistency check. Models predict that increasing *g*_m_ on its own should have a modest positive impact on CO_2_ assimilation rates, as observed here (Clarke *et al*., 2022). Further analysis showed that the observed increases in *A* and apparent *V*_c,max_ were entirely explained by the observed increase in *g*_m_ (Figure 5).

Mesophyll conductance is the net effect of several barriers to CO_2_ diffusion and is influenced by several aspects of leaf anatomy. Thus, it is important to identify the main drivers of the observed increases in *g*_m_. To investigate this, measured values of the anatomical traits *f*_ias_, *T*_mes_ and *S*_c_ were used to calculate CO_2_ conductance across the intercellular airspace, cell wall and membrane (Figure 1). These results indicated that CGR3 expression increased *g*_m_ via increased CO_2_ conductance across the cell wall, without any change in conductance through the intercellular air space or beyond the wall to Rubisco (Figure 1). Likewise, increased *g*_cw_ may be due to decreased *T*_cw_, increased effective porosity or both. Effective porosity estimated from *g*_cw_ and *T*_cw_ shows that both effects are required to explain the increased *g*_cw_ (Figure 1). It will remain difficult to verify this until methods are established to directly measure effective porosity and each of the conductances within *g*_m_, such as a recently published method for quantifying *g*_ias_ (Márquez *et al*., 2023). There were other changes in the leaf. LMA was slightly decreased (although this was not significantly different from WT), which would be expected with a slightly smaller investment in cell wall, which can represent 70% of leaf dry mass (Ye *et al*., 2020). Altering leaf anatomy, more specifically mesophyll cell geometry and packing, could influence the distribution of light within the leaf and, therefore, change leaf absorptance (Ren *et al*., 2019). Although we did not measure the absorptance of these plants, no obvious differences were observed in cell geometry, chloroplast thickness or chlorophyll content (Table S2). In addition, our variable *J* method makes a best‐fit estimate for *τ*, defined by *τ* = absorptance × *β*, where *β* is the fraction of absorbed light energy directed to photosystem II. The value of *β* is difficult to experimentally measure and it is often assumed to be 0.5. Variations in *τ* are expected to be mostly due to variations in absorptance, since it is unlikely that *β* has been altered in the transgenic plants. No significant differences in *τ* were observed, indicating that leaf absorptance was likely unchanged across genotypes (Table S3). Cell wall composition analysis showed no differences in total pectin or other cell wall components, consistent with results seen in *Arabidopsis*, suggesting that increases in cell wall effective porosity were due to increased pectin methylation (Kim *et al*., 2015). Glycome profiling of the cell wall could be used to gain more insight into changes in cross‐linking within the wall by CGR3 expression and how these alterations affect porosity.

Genetic manipulations can affect multiple traits, making it difficult to identify transgenic manipulations that alter *g*_m_ without pleiotropic changes. The few studies successful in increasing *g*_m_ and *A* have either altered additional traits such as true *V*_c,max_, or are unclear about whether these have been altered, making it difficult to determine if *g*_m_ alone can increase photosynthetic rates (Flexas *et al*., 2006; Gong *et al*., 2015; Lehmeier *et al*., 2017; Xu *et al*., 2019). Here, we do not observe any changes to the true *V*_c,max_, that is, that derived from the response of *A* to *C*_c_ (Figure 5). Decreasing thickness and increasing porosity of the cell wall could be expected to alter mechanical strength of the plant, plant hydraulics or stomatal function. In the greenhouse and field, there was no observable evidence of any effect on pest damage or plant form. Stomatal density on the adaxial or abaxial leaf surfaces was unchanged, and there was no significant effect on *g*_sw_ (Table S2; Figure S3). Thus, cell wall thickness and porosity have been successfully modified to increase *g*_m_ and *A* without introducing any apparent unintended pleiotropic effects.

Despite the significant increase in *A*, no corresponding change in biomass was found in the field. Here, *CGR3* was fused with the *A. thaliana* ubiquitin 10, and so the cell wall changes were likely throughout the plant. It is conceivable that the use of the constitutive promoter increased metabolic costs in plant tissue other than leaves, constraining any increase in plant growth. In fact, it has been shown that tissue‐specific or inducible promoters can be more advantageous than constitutive promoters (Su and Wu, 2004). Future experiments would ideally use leaf mesophyll‐specific promoters. Mesophyll‐specific expression of cell wall properties has been obtained using the Rubisco small subunit 1a (*RBCS1A*) promoter (Zhang *et al*., 2021).

A major challenge in increasing crop productivity for food security is the availability of water (Ort and Long, 2014). Agriculture accounts for over 70% of water use, and with rising population and climate change, there is little opportunity to gain further water for agricultural use (Liu *et al*., 2022). The air in the sub‐stomatal air spaces of leaves is close to water vapor saturation when the outside air has high water vapor pressure (Wong *et al*., 2022). This means that while increased stomatal conductance will result in increased water loss, increased mesophyll conductance should not have a direct effect on water vapor loss from the leaf. Among the several different approaches to increasing photosynthesis to support increased crop productivity, increasing *g*_m_ is exceptional in its potential to allow an increase in carbon gain without increased water loss (Long *et al*., 2015). However, in practice, this has not been observed, as *A* and *g*_sw_ are strongly correlated, although the mechanistic basis of their interdependence is not well understood (Leakey *et al*., 2019). Here, increases in *A* and *g*_m_ in the field‐grown plants were balanced by increases in *g*_sw_ (Figure 4d), and no changes in iWUE were observed. It is possible that drought conditions may alter this interdependence, allowing for increased *g*_m_, *A* and iWUE. If true, increased *g*_m_ may be most beneficial for sustaining carbon assimilation of plants grown in water‐limited environments. Our field plants were subjected to temperatures as high as 35 °C in the field, which would have driven large transpiratory fluxes (Figure S5c); however, the field plants were irrigated. A recent study by Pathare *et al*. showed engineered increases in *g*_m_ resulted in increased biomass of rice plants grown under reduced soil water content but not those subjected to ample water (Pathare *et al*., 2024). Taken together these results suggest that follow‐up studies evaluating the CGR3 overexpression lines under drought stress conditions could be of interest as they may result in improvements in biomass and water use.

Taken together, these results provide a critical proof of concept that increasing *g*_m_ by altering the cell wall is a route for enhancing photosynthetic performance of crops. Specifically, the current study shows modification of thickness and porosity as a viable route to improvements in photosynthesis. Gains in water use efficiency could, therefore, be achieved by combining this increase in *g*_*m*_ with decreased *g*_sw_, maintaining the same rate of CO_2_ assimilation while reducing water loss from transpiration. Several approaches have now been identified to allow an engineered or bred decrease in stomatal conductance (Buckley *et al*., 2019; Franks *et al*., 2015; Głowacka *et al*., 2018; Lawson *et al*., 2014). Stacking increased *g*_m_ with other traits such as increased Rubisco activity also has the potential to further increase photosynthetic efficiency. It will be important to consider that certain engineering strategies will only be viable in specific crop species, such as the one here which only applies to C_3_ dicots. Thus, this work complements previous studies that have modified aquaporins and other aspects of leaf architecture and extends the engineering “toolbox” available for controlling *g*_m_ to further increase photosynthetic efficiency and growth needed to sustainably increase food production.

## Experimental procedures

### Plasmid design and assembly

Vector design and construct assembly followed the genetic syntax of the Phytobrick standard (Patron *et al*., 2015) and Loop assembly by Pollak *et al*. (2019). All required genetic modules were domesticated for BpiI, BsaI and SapI prior to *de novo* synthesis through TWIST Bioscience. The nucleotide sequence of *Arabidopsis thaliana* Cotton Golgi‐related 3 (CGR3; AT5G65810.1) was extracted from the *Arabidopsis* Information Resource (TAIR10) (Berardini *et al*., 2015) and codon optimized for *N. tabacum* (IDT™ Codon Optimization Tool). Original and codon‐optimized CGR3 sequences can be found in Data S1. CGR3 was fused with the *A. thaliana* ubiquitin 10 (AT4G05320.2) promoter, including the 5′UTR and first intron, a C‐terminal 1× FLAG tag and the *A. thaliana* heat shock protein 18.2 3′UTR and terminator (AT5G59720). The CGR3 cassette was combined with a CaMV35S:BAR selection marker and cloned into the pCsB acceptor backbone (Addgene #136068) prior to electroporation into *A. tumefaciens* C58C1. Complete plasmid sequence was verified using next‐generation sequencing.

### Plant transformation

*Nicotiana tabacum* cv. Samsun leaf‐disc transformation was performed following (Wang, 2015). The following minor modifications were made to the protocol: fully expanded leaf surfaces were submerged in a sterilization solution for 10 min. Sterilized leaf discs were rinsed with sterile de‐ionized water and cultured in the pre‐culture medium. Explants were further incubated at 24 °C, with a 16‐h light period for 48 h. *A. tumefaciens* C58C1 containing the target vector was grown overnight to an OD600 of 1.0–1.5 in YEP. Leaf discs were then co‐cultivated on fresh pre‐culture medium for 48 h. After 48 h, leaf discs were transferred to a selection medium and incubated under a 16‐h light period, followed by sub‐culturing every 3–4 weeks. Once the shoots reached around 8–10 cm, they were transferred to the rooting medium. All media and solution components are described in Methods S1. Established plants were transferred to soil for acclimatization and maturation in the greenhouse after 3–4 weeks.

### Plant growth – Greenhouse conditions

T2 homozygous seeds from three independent transgenic events and WT *N. tabacum* cv. ‘Samsun’ seeds from which the transgenics were derived and of the same harvest date were germinated on BM6 growing medium (BM6 All‐Purpose, Berger) under greenhouse conditions. Ten days after germination, seedlings were transplanted to 9 cm × 9 cm plastic potting trays. After approximately 2 weeks, plantlets were transplanted to 3.8‐L plastic pots (400C; Hummert International) filled with BM6 growing medium supplemented with 15 cm^3^ of 15–9‐12 (N‐P‐K) granulated slow‐release fertilizer (Osmocote Plus; ICL‐Growing Solutions). Plants were grown under natural illumination with ~300 μmol m^‐^^2^ s^‐1^ of supplemental light at 28 °C/12‐h days and 22 °C/12‐h nights. Chlorophyll content was measured using a SPAD chlorophyll meter (502; Spectrum Technologies). Leaf mass per area (LMA) was measured from six leaf discs each ~1.3 cm^2^ which were dried until constant weight and weights were recorded. After approximately 9 weeks of growth, tobacco plants were harvested. At harvest leaf number, plant height (equal to stem length) and leaf area (LI‐3100C area meter; LI‐COR) were measured. Stem and leaves were dried to a constant weight at 60 °C and dry weights were obtained.

### Transcript and protein expression

Plants were grown under controlled environment greenhouse conditions described above or field conditions described in the following section. Four leaf discs (each ~1.42 cm^2^) were sampled from the youngest fully expanded leaf of 9‐week‐old plants between 11:30 h and 13:30 h, flash frozen in liquid nitrogen and stored at −80 °C until processed. Tissue was disrupted and homogenized (TissueLyser Universal Laboratory Mixer‐Mill disruptor 85 210; QIAGEN) at 20 Hz for one and a half minutes twice, submerging cassettes in liquid nitrogen before each run. mRNA was extracted using the NucleoSpin RNA Plant Kit (Macherey‐Nagel 740 949) modified to increase the first RA3 buffer wash to 650 μL and an additional 400‐μL RA3 buffer wash. RNA quantity and quality were assessed by NanoDrop™ One/OneC (Thermo Fisher Scientific). cDNA was synthesized using the SuperScript™ III First‐Strand Synthesis System (Invitrogen) with random hexamers and 8 μL of RNA.

qPCR was conducted in a 20‐μL reaction of SsoAdvanced Universal SYBR Green Supermix (Bio‐Rad), dilute cDNA, and 500 nmol of each primer and annealing temperature of 59 °C on a CFX Connect Real‐Time PCR Detection System (Bio‐Rad) at 95 °C for 2 min followed by 40 cycles of 95 °C for 15 s and 59 °C for 30 s. Calibrated normalized relative quantities (CNRQ) were calculated using qBase+ software v.3.2 (CellCarta) based on the expression of two reference genes, actin and GAPDH. Primers were designed according to MIQE guidelines (Bustin *et al*., 2009). Primer linear range and efficiency were determined by qPCR on pooled concentrated cDNA from four plots serial diluted by 1:3. Primer efficiencies were between 100 and 103% with a linear range between 0.15 and 333 ng. See Table S1 for primer sequences used in this study.

Total protein was extracted from four leaf discs (each ~1.42 cm^2^) collected and ground as described above. Tissue was mixed with 1× protein buffer (2.5% BME (v/v), 2% SDS (w/v), 10% glycerol (v/v), 0.25 M Tris HCl (pH 6.8)), heated to 95 °C for 5 min and the quantity expressed per unit leaf area. Proteins were separated on 15‐well, 4%–20% Mini‐PROTEAN® TGX™ Precast Protein Gel (Bio‐Rad) and transferred onto polyvinylidene difluoride membranes (Bio‐Rad) using the TransBlot®Turbo™ Transfer System (Bio‐Rad) using the fast TGX protocol. Anti‐FLAG (F7425‐.2MG; Sigma‐Aldrich) and anti‐actin (AS13 2640, Agrisera) primary antibodies were incubated at a 1:5000 dilution overnight at 4 °C in phosphate‐buffered saline with 1% non‐fat dry milk (w/v) and 0.1% Tween‐20. Membranes were incubated with IRDye® 800CW donkey anti‐rabbit IgG secondary antibody (LI‐COR) at a 1:10 000 dilution at room temperature for 1–2 h. Immunoblots were imaged at 800 nm using the LI‐COR Odyssey CLx Infrared Imaging System (LI‐COR).

### Microscopy and anatomical measurements

Leaf tissue was collected from the interveinal region of the youngest fully expanded leaves and fixed in 2% glutaraldehyde (Electron Microscopy Sciences, EMS) and 2.5% paraformaldehyde (Ted Pella Inc). Fixed tissue was stored at 4 °C in the dark until being processed for light and transmission electron microscopy (TEM). Samples were post‐fixed in 2% osmium tetroxide (EMS) and potassium ferrocyanide (Mallinkckrodt Baker Inc) and then stained overnight in 7% uranyl acetate at 4 °C. A graded series of ethanol, ending in 100% ethanol was used to dehydrate the tissue, followed by 100% acetonitrile. The tissue was then infiltrated with 1:1 acetonitrile to Lx112 epoxy mixture (Ladd, Inc), 1:4 and then pure epoxy before hardening at 80 °C overnight. For light microscopy, blocks were trimmed and sectioned at 0.35 microns, stained with toluidine blue and basic fuchsin and viewed with a stereo microscope (BH2, Olympus) coupled with an ocular digital camera (AMT). For electron microscopy, blocks were sectioned at 60−90 nm for electron microscopy and viewed at 75KV where plate film was scanned in at 3200 dpi (H600; Hitachi).

Light micrographs were used to measure the length of mesophyll cells exposed to intercellular airspace (*L*_mes_), the length of chloroplast exposed to intercellular airspace (*L*_c_) and the width of each section measured (*W*). Mesophyll surface area exposed to intercellular airspace (*S*_mes_) and chloroplast surface area exposed to intercellular (*S*_c_) were calculated using equations 4 and 6 from Evans *et al*. (1994).

At least three non‐overlapping fields of view were randomly selected to provide technical replicates, which were averaged to provide a single value for each of the four biological replicates for each genotype. Transmission electron micrographs were used to measure mesophyll cell wall thickness. Ten non‐overlapping fields of view were measured from each of the four biological replicates per genotype. For each image (technical replicate), the area of the cell wall divided by the length was used to calculate cell wall thickness. A total cell wall length of approximately 6500 nm was measured per genotype. This accounts for small variations in thickness along the cell wall. Technical replicates were averaged to provide a single value for each biological replicate (four per genotype). All measurements were made using the freehand area and line selection tools from ImageJ (National Institutes of Health).

### Leaf and cell wall composition analysis

Fully expanded leaves with midrib excised were flash frozen in aluminium foil packets in liquid nitrogen before storage at −80 °C. Three to six grams of tissue were lyophilized to a steady‐state weight. Lyophilized tissue was ground for 15 min at 1200 rpm on a Genogrinder 2010 (*SPEX*) using two 4‐mm stainless steel grinding beads. Total sugars were extracted from 100 to 200 mg of ground, dried tissue by incubation in 80% ethanol at 80 °C for 20 min with decantation six times (Amaral *et al*., 2007). Ethanol extracts were treated with activated charcoal to remove compounds such as lactic acid, sugar alcohols and alcohol‐soluble pigments which can interfere with the reaction and lead to overestimations of sugar content. Total sugar as glucose was measured using the sulfuric‐phenol microplate assay as described in Kondo *et al*. (2021). The protocol was modified to change the heat treatment to 90 °C in a water bath for 5 min. Sugar extract absorbance at 490 nm was measured in triplicate on a Synergy HI Microplate spectrophotometer (Biotek) against a 5–25 μg of glucose standard curve.

After ethanol extraction, the remaining pellet was washed with 1:1 chloroform:methanol (v/v), followed by acetone, and dried overnight at 35 °C. The pellet was subjected to three rounds of digestion by 500 μL of 120 U/mL α‐amylase *Bacillus licheniformis* (Neogen) in 10 mM, pH 6.5 MOPS buffer at 75 °C for 30 min (Amaral *et al*., 2007). The enzyme was deactivated by heating at 99 °C for 10 min. After centrifugation at 13 000 ***g*** for 10 min, 800 μL of supernatant was quantitatively transferred and subjected to two rounds of digestion by 500 μL of 30 U/mL amyloglucosidase *Aspergillus niger* (Neogen) in 100 mM, pH 4.5 acetate buffer at 50 °C for 30 min (Amaral *et al*., 2007). Total starch as glucose was measured by D‐Glucose GOP‐POD microplate assay (nzytech). The pellet from the α‐amylase MOPS digestion was decanted, washed with water twice, and acetone once. The acetone was removed using a Speed Vac Concentrator (Thermo Fisher Scientific) to steady‐state weight resulting in the cell wall alcohol‐insoluble residue (AIR).

In triplicate, 2–3 mg of AIR was digested in 375 μL of 2 M trifluoroacetic acid (TFA) at 121 °C for 90 min (Foster *et al*., 2010). Supernatant was removed and analysed for TFA soluble hemicellulose by the sulfuric‐phenol microplate assay described previously and for pectin as D‐Galacturonic acid per Bethke and Glazebrook (2019) with minor modifications. The addition of 2 mg/mL prepared m‐hydroxydiphenyl reagent was reduced to 10 μL per well and measured absorbance at 525 nm in triplicate on a Synergy HI Microplate spectrophotometer (Biotek) against a 6.25–200 nmol of D‐(+)‐galacturonic acid monohydrate (AAJ6628214; Thermo Fisher Scientific) standard curve.

Cellulose and non‐soluble hemicellulose (primarily glycan) were digested with sulfuric acid as described in Foster *et al*. (2010) and quantified as glucose by the sulfuric‐phenol microplate assay described previously against a 2–12 μg of glucose standard curve. Detailed protocol is available at protocols.io. https://doi.org/10.17504/protocols.io.3byl4q6jzvo5/v1.

### Stomatal density

Adaxial and abaxial stomatal impressions of approximately 2 cm^2^ were made on the youngest fully expanded leaf of greenhouse‐grown plants as described previously (Weyers and Johansen, 1985). Six plants per genotype were sampled. Four images were obtained per impression using the Axio Imager A1 microscope (Zeiss) equipped with the Zeiss AxioCam HrC digital camera, AxioVision software version 4.9.1.0 (Zeiss) and a 20×/0.5 objective (EC Plan‐Neofluar420350‐9900). All whole stomata and partial stomata on the left and top borders of the image were counted using Cell Counter Plugin (https://imagej.net/ij/plugins/cell‐counter.html) in ImageJ (Schneider *et al*., 2012) and used to calculate stomatal density.

### Estimating mesophyll conductance using carbon isotope discrimination coupled with leaf gas exchange

The LI‐COR 6800 gas exchange system (LI‐COR Environmental) was coupled to a tunable‐diode laser absorption spectroscope (TDLAS model TGA 200A; Campbell Scientific) to measure online carbon isotope discrimination (Tazoe *et al*., 2011; Wang *et al*., 2022). The TDL was connected to the LI‐6800 reference and sample air streams using the ports on the back of the sensor head. N_2_ and O_2_ were mixed using mass flow controllers (OMEGA Engineering Inc.) and spilt into multiple lines to use as CO_2_ free air. One line was used to zero the TDL throughout the measurements. Two lines supplied the inlets of two gas exchange systems to make measurements at 2% O_2_. The final line was diluted with a 10% CO_2_ gas cylinder to produce three different CO_2_ concentrations (60, 300 and ~1000 ppm CO_2_) of the same isotopic signature and used to calibrate the ^13^CO_2_ signal.

The measurements cycled through nine gas streams in the following sequence: calibration zero, calibration points 60, 300 and 1000 ppm CO_2_, NOAA calibration of δ^13^C composition (NOAA Global Monitoring Laboratory), LI‐COR 6800 #1 reference and leaf chamber air streams and LI‐COR 6800 #2 reference and leaf chamber air streams. Each step had a duration of 20 s and measurements were averaged over the last 10 s to produce a single data point.

Gas exchange measurements were made under the following conditions: light intensity of 1800 μmol m^‐^^2^ s^‐1^, leaf temperature of 25 °C, leaf vapor pressure deficit of 1.3 kPa, 2% O_2_ and 400 μmol mol^‐1^ CO_2_. Two‐per cent oxygen was used to minimize photorespiration. Once steady‐state CO_2_ assimilation and stomatal conductance were reached, the gas exchange system was set to auto‐log at 180‐s intervals over the course of 30 min. After the program was completed, the light was turned off and dark respiration rate was measured on plants after >30 min in the dark.

The combined gas exchange and TDLAS data were processed and analysed using PhotoGEA, an R package for photosynthetic gas exchange analysis (Lochocki, 2023). This process generally followed the steps described in the “Analyzing Mesophyll Conductance Data” article included with PhotoGEA, which is also available online at the PhotoGEA documentation website: https://eloch216.github.io/PhotoGEA/.

Within each TDL cycle, correction factors derived from the five calibration tanks were used to obtain calibrated dry air [^12^CO_2_] and [^13^CO_2_] in the air streams entering and exiting each LI‐COR leaf chamber. The isotopic composition (δ^13^C) of each air stream was calculated using equation 4 from Ubierna *et al*. (2018). Timestamps and TDL valve numbers were then used to pair each TDL measurement with its corresponding gas exchange log entry, enabling the calculation of the observed photosynthetic ^13^CO_2_ discrimination (Δ^13^C) and the ternary gas correction factor (*t*) using equations 5 and 9 from Ubierna *et al*. (2018). The CO_2_ compensation point in the absence of day respiration (Γ*) was calculated from [O_2_] and leaf‐temperature‐dependent O_2_ and CO_2_ solubilities assuming a Rubisco specificity of 97.3 M M^−1^ (Walker *et al*., 2013). Finally, mesophyll conductance to CO_2_ diffusion (*g*_mc_) was calculated using equations 13 and 22 from Busch *et al*. (2020), which assume that mitochondrial respiration is isotopically disconnected from the Calvin–Benson–Bassham cycle. The effective isotopic fractionation due to day respiration (e*) was calculated using equation 19 from Busch *et al*. (2020) rather than equation 20, because values of $\Delta_{\mathrm{obs}}^{\text{growth}}$ were not available; however, this should have minimal impact due to the low [O_2_] used for these measurements.

### Plant growth – Field conditions

Seeds from homozygous T2 single insertion events (CGR3‐8, CGR3‐10 and CGR3‐14) and WT seeds from the same harvest date were sown in the greenhouse on May 16th 2022 (DOY 136). After 10 days, seedlings were transplanted to floating trays as described in Kromdijk *et al*. (2016). Plantlets were transplanted to the University of Illinois Energy farm field site (40.11° N, 88.21° W, Urbana, IL) on June 10th 2022 (DOY 161). The field was prepared 1 week prior to transplant as described previously (Kromdijk *et al*., 2016).

The field experiment used a randomized block design with six blocks. Each block consisted of four rows of 10 plants per genotype in a north–south (N‐S) orientation, with plants spaced 61 cm apart (Figure S5a). Each block contained one WT row. In addition, one border row of WT plants surrounded the perimeter of the six experimental blocks. Plants were irrigated as needed using parallel drip irrigation lines (DL077; The Drip Store). Weather data were measured with a digital sensor mounted 10 m above ground level at the same field site (ClimaVUE50; Campbell Scientific, Figure S5b,c).

Plants were harvested on July 21st 2022 (DOY 202). At harvest leaf number, plant height (equal to stem length) and leaf area (LI‐3100C area meter, LI‐COR) were measured. Harvested material was partitioned into leaf, stem and roots for five randomly selected plants per row. These were dried to a constant weight at 60 °C in custom‐built dying ovens and dry weights were obtained.

### Leaf gas exchange in the field

Photosynthetic gas exchange measurements were performed on the youngest fully expanded leaves on July 9th–10th 2022 (DOY 190–191). CO_2_ response curves (*A*–*C*_*i*_) were measured using a LI6800 infrared gas exchange system with integrated leaf chamber fluorometer (LI‐COR). Leaves were clamped into a 6 cm^2^ gas exchange cuvette and acclimated to the following conditions: light intensity of 1800 μmol m^‐^^2^ s^‐1^, leaf temperature of 28 °C, CO_2_ reference concentration of 400 μmol mol^‐1^ and 60% humidity. CO_2_ responses were initiated when rates of CO_2_ assimilation and stomatal conductance stabilized to a steady state (~20 min). Response curves were measured with the following sequence of reference [CO_2_]: 400, 300, 200, 150, 75, 50, 20, 400, 400, 500, 600, 800, 1000, 1200, 1500 and 1800 μmol m^‐^^2^ s^‐1^. Measurements were logged 3–5 min after each new [CO_2_] step. Fluorescence measurements were made at each step using the multi‐phase flash fluorescence protocol with a saturating flash of 10 000 μmol m^‐^^2^ s.

Apparent maximum Rubisco carboxylation rates (*V*_*c*,max_) at 25 °C were estimated using the *fit_c3_aci* function from the PhotoGEA R package (Lochocki, 2023), which fits measured CO_2_ response curves with the Farquhar–von Caemmerer–Berry (FvCB) model, including limitations from triose phosphate utilization (TPU) (von Caemmerer, 2000). Temperature scaling of key parameters (*K*_*C*_, *K*_*O*_, Γ*, *V*_*c*,max_, *J* and *R*_*d*_) was modelled using Arrhenius factors (Sharkey *et al*., 2007) and mesophyll conductance was set to infinity (equivalent to setting *C*_*c*_ = *C*_*i*_). During the fits, an optimization algorithm is used to choose values of the four unknown FvCB model parameters (*V*_*c*,max_, *J* and *R*_*d*_ at 25 °C and the maximum rate of TPU, *T*_*p*_) that produce the best agreement between the modelled and measured CO_2_ assimilation rates.

### Estimating mesophyll conductance using variable *J*

*C*_*c*_, *g*_mc_ and the true *V*_*c*,max_ were estimated from gas exchange measurements made in parallel with chlorophyll fluorescence measurements using the “Variable *J*” fitting method as implemented in the *fit_c3_variable_j* function from the PhotoGEA R package (Lochocki, 2023). In this method, net CO_2_ assimilation (*A*_*n*_) is modelled by (1) calculating *g*_mc_ and *C*_*c*_ from the incident photosynthetically active photon flux density (*Q*_in_), the measured operating efficiency of photosystem II (*φ*_PSII_) and the measured *A*_*n*_, and then (2) using the calculated *C*_*c*_ as an input to the FvCB model (Harley *et al*., 1992; Moualeu‐Ngangue *et al*., 2017). There are five unknowns in the equations used to model *A*_*n*_: *τ* (a proportionality factor that relates *Q*_in_ and φ_PSII_ to the fluorescence‐based estimate of the RuBP regeneration rate) and the four FvCB model parameters (*V*_*c*,max_, *J* and *R*_*d*_ at 25 °C and *T*_*p*_). During the fits, an optimization algorithm is used to choose values of these unknowns that produce the best agreement between the measured and modelled *A*_*n*_. Once these parameter values have been found, values of *C*_*c*_ and *g*_mc_ are also immediately known.

### Estimation of effective porosity

The cell wall effective porosity (*p* / *τ*) can be determined from the cell wall conductance to CO_2_ diffusion (*g*_cw_) provided the cell wall thickness *T*_cw_ is known (Ellsworth *et al*., 2018). In turn, *g*_cw_ can be estimated from *g*_mc_ by accounting for the effect of other known barriers to CO_2_ diffusion (specifically, the intercellular airspace, the plasma membrane and the chloroplast envelope) (Ellsworth *et al*., 2018; Xiong, 2023). Here, we use this approach to calculate *p*/*τ* from measured values of *g*_mc_, *f*_ias_, *T*_cw_, *T*_mes_ and *S*_*c*_. Overall, our method is similar to the one used in Ellsworth *et al*. (2018) but differs by including the conductance across the intercellular airspace and a membrane conductance enhancement factor as in Xiong (2023). For details of the calculations, see Methods S2.

### Statistical analysis

Normality of the data was tested with Shapiro–Wilk's test, and homoscedasticity with Brown–Forsythe test. If criteria for normal distributions and equal variance were met, one‐way ANOVA followed by Dunnett's *post hoc* test for transgenic mean comparison against the WT control was performed. Data were considered significant at *P* < 0.05 and marginally significant at *P* < 0.1. If criteria for normality were violated, Wilcoxon's non‐parametric test was applied. If criteria for equal variance were violated, Welch's ANOVA followed by Games‐Howell *post hoc* test was applied. Analysis of field growth traits (Figure 6) was performed using a randomized block design with six blocks. Tests used are indicated in the figure or table legend. Correlations between 1/*g*_m_ and *T*_cw_, and *g*_m_ and effective porosity were evaluated using Pearson's correlation coefficient. Jmp pro version 17.0.0 software was used for all statistical analyses.

## Conflict of interest

The authors declare no conflicts of interest.

## Author contributions

CES and SPL designed the experiments. BEH assembled the construct and supervised the generation of the transgenic tobacco lines. SSS set up the TDL and maintained the carbon isotope discrimination equipment. LD measured gene expression, cell wall composition and stomatal density. EBL calculated effective porosity, *g*_ias_, *g*_cw_ and *g*_mem_, and developed the PhotoGEA data‐processing R package used to analyse all gas exchange and carbon isotope discrimination data. CES participated in all experiments and analysed the data. CES and SPL wrote the manuscript with contributions from all authors.

## Supporting information


**Data S1** Codon optimized sequence of AtCGR3.
**Figure S1** Gene expression in field‐grown CGR3 and WT lines.
**Figure S2** Sugar and starch content of greenhouse‐grown tobacco plants.
**Figure S3** Stomatal density of greenhouse‐grown tobacco plants.
**Figure S4** Plant growth traits in greenhouse‐grown tobacco plants.
**Figure S5** Tobacco field experimental design and weather conditions.
**Figure S6** Correlation of CO_2_ assimilation to stomatal and mesophyll conductance.
**Methods S1** Plant transformation culture media and solutions components.
**Methods S2** Details for estimation of effective porosity.
**Table S1** qPCR primer information.
**Table S2** Summary of leaf gas exchange combined with carbon isotope discrimination, cell wall composition, leaf mass per area (LMA) and chlorophyll content (SPAD value) of greenhouse‐grown plants.
**Table S3** Summary of harvest measurements from field‐grown plants.

## Data Availability

The data that support the findings of this study are openly available in CGR3‐tobacco‐2024 at https://github.com/ripeproject/CGR3‐tobacco‐2024.
