# Supplementary material for: Greater mesophyll conductance and leaf photosynthesis in the field through modified cell wall porosity and thickness via AtCGR3 expression in tobacco
Source: Plant Biotechnol J. 2024 Apr 30;22(9):2504–17. doi: 10.1111/pbi.14364 (PMC11331791; doi:10.1111/pbi.14364)
Supplement: Supplementary file 1 — Data S1 Codon optimized sequence of AtCGR3. Figure S1 Gene expression in field‐grown CGR3 and WT lines. Figure S2 Sugar and starch content of greenhouse‐grown tobacco plants. Figure S3 Stomatal density of greenhouse‐grown tobacco plants. Figure S4 Plant growth traits in greenhouse‐grown tobacco plants. Figure S5 Tobacco field experimental design and weather conditions. Figure S6 Correlation of CO2 assimilation to stomatal and mesophyll conductance. Methods S1 Plant transformation culture media and solutions components. Methods S2 Details for estimation of effective porosity. Table S1 qPCR primer information. Table S2 Summary of leaf gas exchange combined with carbon isotope discrimination, cell wall composition, leaf mass per area (LMA) and chlorophyll content (SPAD value) of greenhouse‐grown plants. Table S3 Summary of harvest measurements from field‐grown plants. [file PBI-22-2504-s001.pdf]

Supporting Information

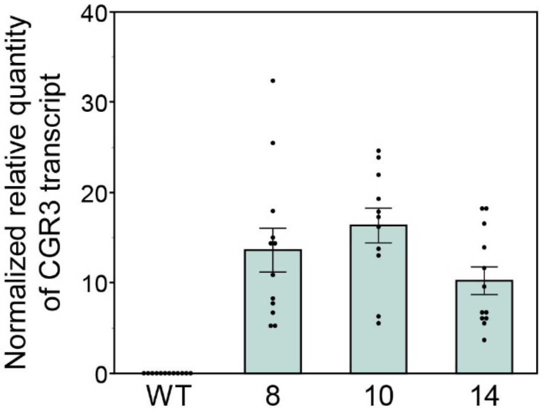

Figure S1| Gene expression in field grown CGR3 and WT lines.

qPCR analysis of AtCGR3 gene expression in three independent transgenic events (8, 10, and 14). Values are shown as the mean ± SEM (n = 12).

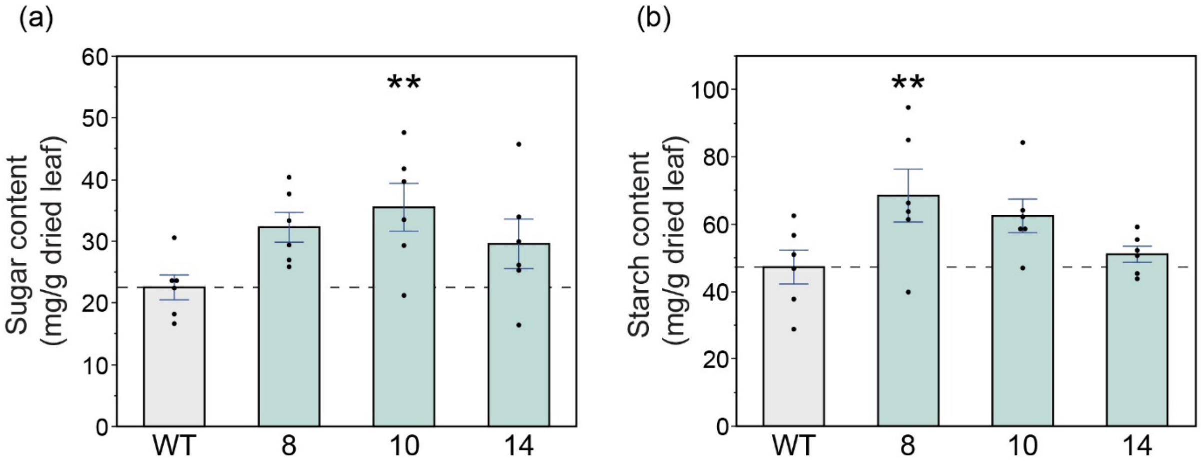

Figure S2| Sugar and starch content of greenhouse grown tobacco plants.

Total **(a)** sugar and **(b)** starch content as glucose measured in dried leaf tissue. Values are shown as the mean ± SEM (n = 6). Asterisks indicate significant differences between WT and the CGR3 transgenic line (**$P < 0.05$); one-way ANOVA, Dunnett's post hoc test.

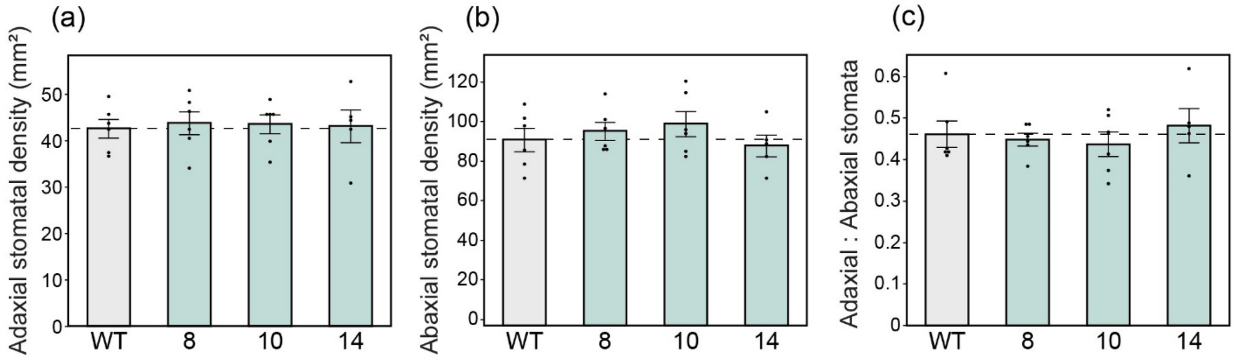

Figure S3| Stomatal density of greenhouse grown tobacco plants.

(a) Stomatal density on adaxial leaf surface, **(b)** stomatal density on abaxial leaf surface, and **(c)** adaxial to abaxial stomatal density ratio. Values are shown as the mean $\pm$ SEM (n =6). No significant differences, one-way ANOVA, Dunnett's post hoc test.

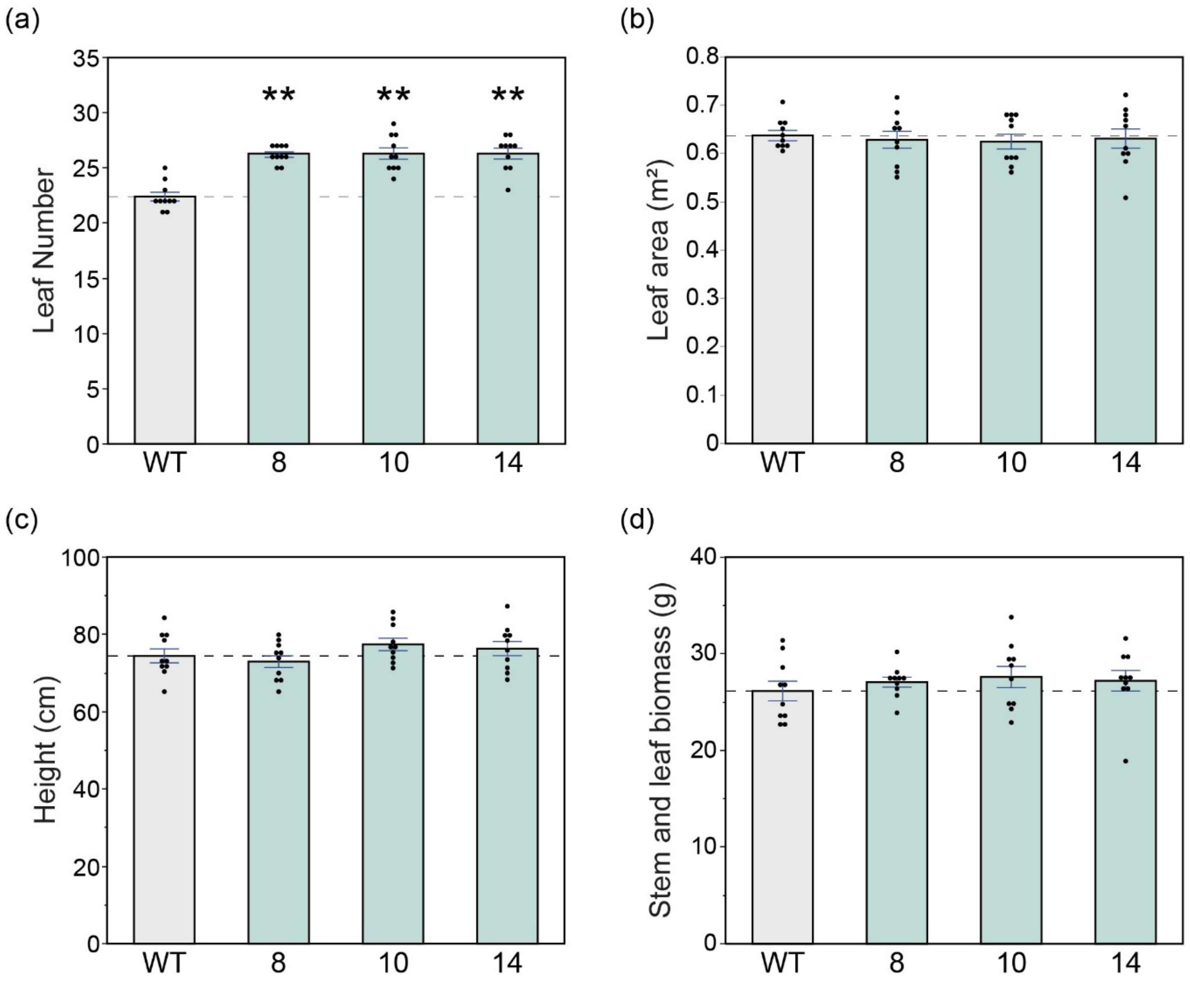

Figure S4| Plant growth traits in greenhouse grown tobacco plants.

(a) Leaf number, **(b)** leaf area, **(c)** plant height and **(d)** biomass (sum of leaf and stem dry weights). Values are shown as the mean ± SEM (n = 10). Asterisks indicate significant differences between WT and the CGR3 transgenic line (**P < 0.05); (a) Wilcoxon's non-parametric test; (b-d) one-way ANOVA, Dunnett's post hoc test.

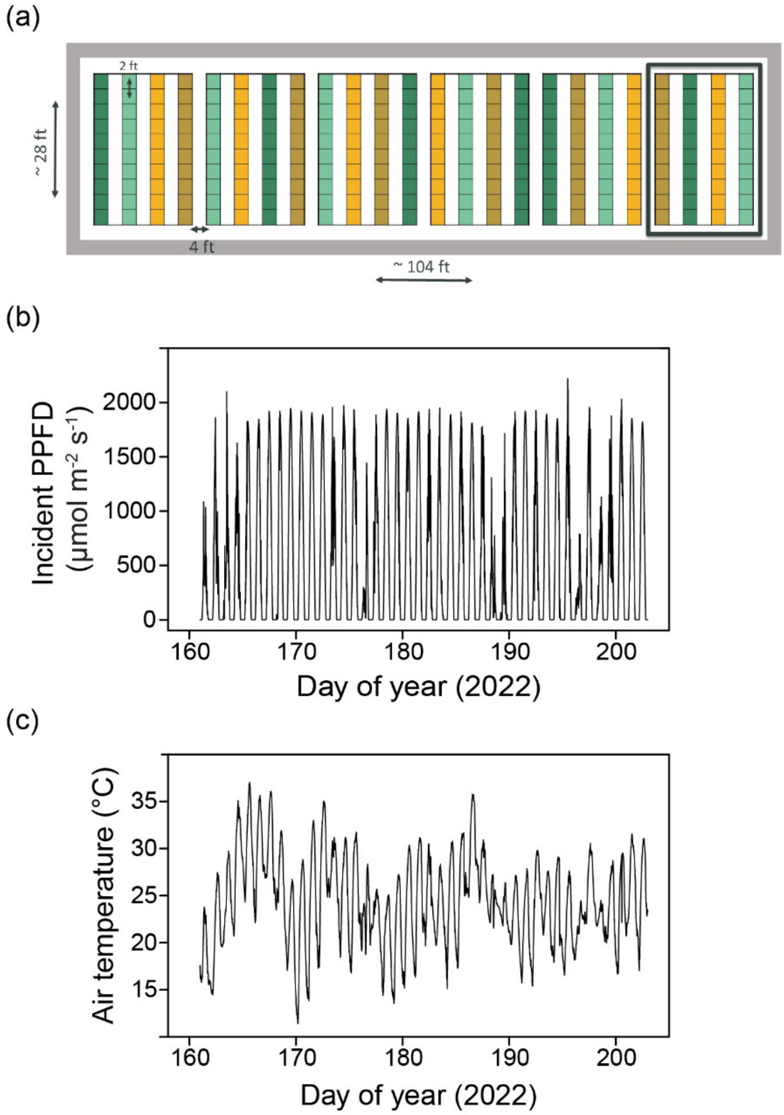

Figure S5| Tobacco field experimental design and weather conditions.

(a) Schematic of field experimental set up. Genotypes were randomly assigned to one of four possible row positions in each of the six blocks. **(b)** Light intensity from June 10th (DOY 161 - date of tobacco transplant into the field) until July 21st, 2022 (DOY 202 - date of tobacco plant harvest). **(c)** Air temperature from June 10th until July 21st, 2022 (DOY 161 to 202). Data are shown as averages over the preceding hour.

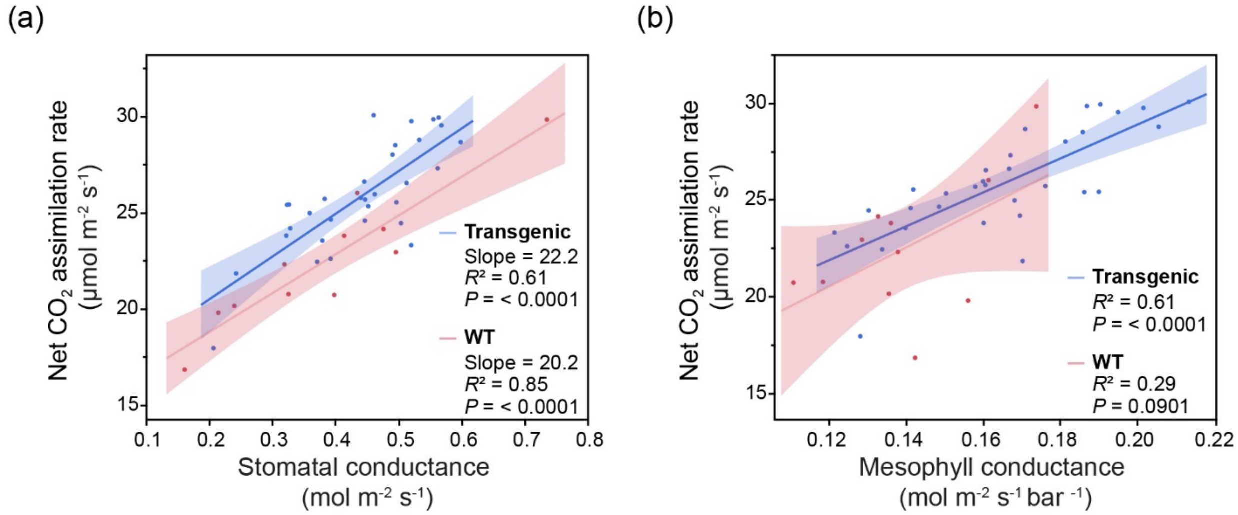

Figure S6| Correlation of CO_2 assimilation to stomatal and mesophyll conductance in field-grown plants.

The relationship between CO_2 assimilation and **(a)** stomatal conductance, or **(b)** mesophyll conductance. Each point corresponds to the values measured from one plant. Measurements from transgenic (blue) and WT (red) plants are shown. The solid lines represent linear regressions from the data points calculated using Pearson's coefficient of correlation. The blue and red areas are 95% confidence intervals of the regression lines.

Table S1 qPCR primer information

Primer name	Sequence	Amplicon length
CGR3_F	GTGATCGTCTCCGATGCACT	80
CGR3_R	CTTGCAACACGAGCCAGTTC	
GAPDH_F	CAA CCC CTA ACG TCT CGG TC	92
GAPDH_R	AGCAGCCTCCCTAAATGCAG	
Actin_F	CCT CAC AGA AGC TCC TCT TAA TC	137
Actin_R	ACA GCC TGA ATG GCG ATA TAC	

Table S2. Summary of leaf gas exchange combined with carbon isotope discrimination measurements and anatomical measurements not presented in main data, cell wall composition, leaf mass per area (LMA) and chlorophyll content (SPAD value) of greenhouse grown plants. Values are shown as the mean ± SEM. Values significantly different from WT at $P < 0.05$ are in bold. One-way ANOVA, Dunnett's post hoc test.

Parameter	WT	CGR3 – 8	CGR3 – 10	CGR3 – 14	Sample number (N)
A ($\mu mol\ m^{-2}\ s^{-1}$)	31.37 ± 0.98	**35.64 ± 1.13**	33.45 ± 1.20	32.93 ± 0.81	8-10
$1/g_m$ ($mol\ m^{-2}\ s^{-1}$ bar^{-1})	2.50 ± 0.15	**1.83 ± 0.10**	**2.00 ± 0.16**	**2.04 ± 0.11**	8-10
g_{sw} ($mol\ m^{-2}\ s^{-1}$)	0.537 ±0.023	0.533 ± 0.032	0.489 ± 0.023	0.515 ± 0.020	8-10
S_c	8.28 ± 0.34	8.84 ± 0.15	9.39 ± 0.25	8.57 ± 0.45	4 plants
S_m	9.70 ± 0.26	9.86 ± 0.12	10.66 ± 0.31	9.74 ± 0.50	4 plants
Leaf thickness (µm)	249.4 ± 15.01	241.8 ± 9.25	264.6 ± 11.68	269.6 ± 20.10	4 plants
Chloroplast thickness (µm)	2.46 ± 0.19	2.36 ± 0.15	2.37 ± 0.19	2.37 ± 0.21	4 plants
Chloroplast length (µm)	5.33 ± 0.18	5.30 ± 0.25	5.40 ± 0.14	5.30 ± 0.37	4 plants
Pectin (mg/g AIR)	86.90 ± 7.42	82.92 ± 2.15	68.94 ± 5.83	69.02 ± 12.4	6
Hemicellulose (mg/g AIR)	157.13 ± 6.52	144.18 ± 10.3	137.11 ± 14.8	159.29 ± 5.05	6
Cellulose (mg/g AIR)	54.22 ± 2.19	51.36 ± 5.42	47.59 ± 3.36	64.94 ± 4.37	6
Pectin / (Hemicellulose + Cellulose)	0.412 ± 0.035	0.429 ± 0.025	0.302 ± 0.050	0.308 ± 0.055	6
LMA (g/m^2)	30.27 ± 2.21	27.13 ± 1.81	28.40 ± 2.02	28.89 ± 1.06	5-6
SPAD	55.30 ± 2.47	51.52 ± 2.28	56.57 ± 2.35	52.28 ± 0.756	5-6

Table S3. Tau from Variable *J* fits and summary of harvest measurements from field-grown plants. Values are shown as the mean ± SEM. Values are not significantly different from WT at $P < 0.05$.

Parameter	WT	CGR3 – 8	CGR3 – 10	CGR3 – 14	Sample number (*N*)
Tau	0.590 ± 0.005	0.575 ± 0.011	0.580 ± 0.013	0.596 ± 0.008	10-11
Leaf number	30.9 ± 3.32	31.1 ± 2.79	32.8 ± 4.55	31.3 ± 4.30	6 plots
Leaf weight (g)	29.7 ± 3.21	30.9 ± 3.37	31.5 ± 4.53	33.5 ± 5.55	6 plots
Stem weight (g)	13.8 ± 2.03	15.0 ± 2.46	14.3 ± 3.23	14.6 ± 3.17	6 plots
Root weight (g)	10.6 ± 2.22	11.9 ± 2.89	10.2 ± 3.44	10.4 ± 3.16	6 plots
Above ground biomass (g)	43.4 ± 5.14	45.9 ± 5.78	45.8 ± 7.73	48.0 ± 8.68	6 plots

Methods S1. Plant transformation culture media and solution components.

<u>Sterilization solution</u>
10% (v/v) Bleach (Sodium hypochlorite)
0.02% Tween 20® (v/v) (Sigma-Aldrich P1379)
1 ml/L Plant Preservative Mixture (PPM™, Plant Cell Technology Inc.)

<u>Pre-culture medium</u>
Murashige and Skoog complete medium with vitamins (Phytotech labs M519)
3% D-Sucrose (Phytotech labs S829)
2 mg/L Kinetin (Phytotech labs K750)
1.0 mg/L Indole-3-Acetic Acid (IAA, Phytotech labs I885)
0.2% Gelzan™ (Phytotech labs G3251)
pH adjusted to 5.6 with 1N KOH and made to required volume

<u>Selection medium</u>
Murashige and Skoog complete medium with vitamins (Phytotech labs M519)
3% D-Sucrose (Phytotech labs S829)
2 mg/L Kinetin (Phytotech labs K750)
1.0 mg/L Indole-3-Acetic Acid (IAA, Phytotech labs I885)
25mg/L Vancomycin (Phytotech labs V8370)
400mg/L Timentin (Phytotech labs T869)
2.0 mg/L Phosphinothricin (Gold Bio P-165-250)
0.2% Gelzan™ (Phytotech labs G3251)

<u>Rooting media</u>
Murashige and Skoog complete medium with vitamins (Phytotech labs M519)
3% D-Sucrose (Phytotech labs S829)
400mg/L Timentin (Phytotech labs T869)
2.0 mg/L Phosphinothricin (Gold Bio P-165-250)
0.2% Gelzan™ (Phytotech labs G3251)

Methods S2. Details for estimation of effective porosity

The full path taken by CO_2 from the ambient air to Rubisco includes eight sequential barriers, each of which has an associated conductance g: the boundary layer (g_{bl}), stomata (g_{st}), intercellular airspace (g_{ias}), cell wall (g_{cw}), plasma membrane (g_{pm}), cytosol (g_{cyt}), chloroplast limiting membranes (g_{clm}), and chloroplast stroma (g_{stroma}) (Nobel 2009). By convention, the final six components are often grouped together into a combined mesophyll conductance (g_m). Within the mesophyll components, other groupings can also be formed, such as the liquid phase conductance g_{liq} (consisting of g_{cw}, g_{cyt}, and g_{stroma}) and the membrane conductance g_{mem} (consisting of g_{pm} and g_{clm}) (Evans and von Caemmerer 2013). The cytosol and stroma conductances are difficult to estimate but are expected to be large and similar between genotypes, so they likely play a small role in determining differences in g_m and can be neglected (Ellsworth et al. 2018). Under these assumptions, the mesophyll conductance can be expressed as

$$\frac{1}{g_m} = \frac{1}{g_{ias}} + \frac{1}{g_{cw} \cdot S_c} + \frac{1}{g_{mem} \cdot S_c}, \quad (1)$$

where S_c is the chloroplast surface area appressing intercellular airspace per unit leaf area (m^2 chloroplast m^{-2} leaf), g_m and g_{ias} are expressed on a leaf area basis (mol m^{-2} leaf s^{-1} bar^{-1}), g_{cw} and g_{mem} are expressed on a chloroplast area basis (mol m^{-2} chloroplast s^{-1} bar^{-1}), and $\frac{1}{g_{mem}} = \frac{1}{g_{pm}} + \frac{1}{g_{clm}}$. The factor S_c converts conductances from a chloroplast area basis to a leaf area basis.

The cell wall conductance is determined from its anatomy according to

$$g_{cw} = \frac{p \cdot D_{liq} \cdot K_{CO_2}}{\tau \cdot T_{cw}} \cdot \frac{1}{R \cdot T_{leaf}}, \quad (2)$$

where p is the cell wall porosity (dimensionless), D_{liq} is the diffusivity of CO_2 in water (m^2 s^{-1}), K_{CO2} is the partition coefficient for CO_2 in water (dimensionless), τ is the tortuosity of the CO_2 diffusion path through the cell wall (dimensionless), T_{cw} is the cell wall thickness (m), T_{leaf} is the leaf temperature (K), and R is the ideal gas constant (J k^{-1} mol^{-1}) (Ellsworth et al. 2018). Dividing by $R \cdot T_{leaf}$ converts the conductance from m s^{-1} to mol m^{-2} s^{-1} bar as discussed below. Porosity and tortuosity are difficult to estimate, and can instead be combined into an effective porosity p / τ (Ellsworth et al. 2018). Combining Equations 1 and 2, the effective porosity can be calculated as

$$\frac{p}{\tau} = \frac{T_{cw} \cdot R \cdot T_{leaf} \cdot g_{cw}}{D_{liq} \cdot K_{CO_2}} = \frac{T_{cw} \cdot R \cdot T_{leaf}}{D_{liq} \cdot K_{CO_2}} \cdot \left[\frac{S_c}{g_m} - \frac{S_c}{g_{ias}} - \frac{1}{g_{mem}}\right]^{-1}. \quad (3)$$

This is a useful expression because all factors on the right-hand-side can either be measured or calculated, enabling an estimate of the effective porosity. The conductance in the intercellular airspace can be calculated as

$$g_{ias} = \frac{D_{air} \cdot f_{ias}}{0.5 \cdot T_{mes} \cdot \xi} \cdot \frac{1}{R \cdot T_{leaf}}, \quad (4)$$

where D_{air} is the diffusivity of CO_2 in air, f_{ias} is the fraction of intercellular airspace within the mesophyll, T_{mes} is the mesophyll thickness, and ξ is the tortuosity of the CO_2 diffusion path through the intercellular airspace (Xiong 2023). The membrane conductance can be calculated as

$$g_{mem} = (1 + \epsilon_{mem}) \cdot g_{mem}^{bilayer}, \quad (5)$$

where $g_{mem}^{bilayer}$ is the membrane conductance as estimated from the permeability of a lipid bilayer (given by Equation 5 in (von Caemmerer and Evans (2015)) and ϵ_{mem} is a dimensionless conductance enhancement factor accounting for facilitation processes (Xiong 2023). Here we use $\epsilon_{mem} = 2$, as estimated from the Solanaceae crops (potato and tomato) included in Figure 7d of Xiong (2023).

The diffusivity of CO_2 in water (D_{liq}) can be calculated using Equation 4 from von Caemmerer and Evans (2015). The cell wall thickness T_{cw}, mesophyll thickness T_{mes}, and fraction of intercellular airspace f_{ias} can be determined from microscopy images. The mesophyll conductance g_m can be estimated from combined gas exchange and isotope discrimination measurements. The leaf temperature (25 °C) is known from measurement conditions, and temperatures are converted from °C to K by adding 273 (von Caemmerer and Evans 2015). The values of the remaining parameters were:

- K_{CO2} = 1 (Nobel 2009)
- R = 8.314 J K^{-1} mol^{-1} (von Caemmerer and Evans 2015)
- D_{air} = 1.515 x 10^{-5} m^2 s^{-1} (Xiong 2023)
- ξ = 1.57 m m^{-1} (Xiong 2023)

Care must be taken when calculating g_{cw} and g_{ias} from anatomical parameters. The equations as written in the sources cited calculate these conductances in units of m s^{-1}. Thus, they are intended to be used along with gas concentrations expressed in vapor densities (kg m^{-3}) rather than partial pressures (bar). It can be shown using the ideal gas law that conductances in m s^{-1} can be divided by $R \cdot T_{leaf}$ to convert to the correct units, hence the appearance of this factor in our Equations 2 and 4. (Note that 1 J = 1 Pa m^3 and 1 bar = 10^5 Pa.)

Equation 3 for the effective porosity is similar to the approach taken in Ellsworth et al. (2018), but it also includes the conductance through the intercellular airspaces and a membrane conductance enhancement factor as in Xiong (2023).

Data S1. Codon optimized sequence of AtCGR3

'AtCGR3' is original Arabidopsis CGR3 full length coding sequence (AT5G65810.1)

'AtCGR3_NtCO' is Arabidopsis CGR3 sequence codon optimized for *Nicotiana tabacum*

'.' indicates base remains unchanged

```
Identities:581/777(75%), Strand: Plus/Plus

AtCGR3       1    ATGTCAAGAAGGCAAGTAAGGCGTGTAGGGGATAGTGGAAGCTTCCCATTTGTAGGAGCT  60
AtCGR3_NtCO  1    ..............G..C...........A..CTCC..GTCA.....T..C..T......  60

AtCGR3       61   CTGCATTCAAAATCACGTTCGTCTCCTCTGTTATCAGTTTGCCTTGTTCTCGTGGGAGCA  120
AtCGR3_NtCO  61   T.A..CAGC.....T.....C........C.....C..C..TT.A..A.....T..T..C  120

AtCGR3       121  TGCCTTCTCATTGGTTATGCTTACAGTGGTCCAGGTATGTTCAAAAGTATCAGAGAAGTC  180
AtCGR3_NtCO  121  .....CT.G........C..C...TC...A..T...........G..C.....G.....T  180

AtCGR3       181  AGCAAGATTACAGGTGACTATTCTTGCACAGCAGAAGTTCAAAGAGCCATTCCTATTCTT  240
AtCGR3_NtCO  181  ..........T..C..T.....A.....C..T..G..A...C.T............T.G  240

AtCGR3       241  AAGAGTGCGTATGGAGATAGCATGCGCAAAGTCCTGCACGTGGGTCCTGAAACATGCTCA  300
AtCGR3_NtCO  241  ..A..C..T.....T...TCA.....A..G..TT....T..T..G.....G..T......  300

AtCGR3       301  GTGGTCTCGAGTCTGTTGAATGAAGAAGAGACAGAAGCATGGGGTGTTGAACCATATGAT  360
AtCGR3_NtCO  301  ..A...AGCTCA...C....C..G..........G..T.....C..A.....T..C...  360

AtCGR3       361  GTGGAGGATGCAGACTCTAACTGCAAAAGTCTTTTGCACAAGGGCCTTGTACGTGTGGCT  420
AtCGR3_NtCO  361  ..C..A...........C..T.....GTCA..G.....T..A..T..G.....A..C..A  420

AtCGR3       421  GACATCAAATTCCCTCTTCCTTACCGGTCAAAGTCGTTTTCTCTTGTGATCGTCTCAGAC  480
AtCGR3_NtCO  421  ........G..T..CT.G..A..TA....T.....A........C...........C..T  480

AtCGR3       481  GCTTTGGATTACCTCTCACCCAGGTACCTGAACAAAACTGTGCCTGAACTTGCTCGCGTC  540
AtCGR3_NtCO  481  ..AC.C........G........A...T.A..T..G........C.....G.....T..T  540

AtCGR3       541  GCTTCAGATGGTGTCGTTCTTTTAGCAGGTAACCCTGGTCAACAAAAGGCTAAAGGTGGG  600
AtCGR3_NtCO  541  ..AAGT........A..C...C.T..T..A.....A..A........A.....G.....T  600

AtCGR3       601  GAATTGTCGAAATTTGGACGGCCTGCTAAAATGCGTAGCTCGTCG-TGGTGGATCCGTTT  659
AtCGR3_NtCO  601  ......AGT.........A....C.........A.G...-A..A.C........AA.A..  659

AtCGR3       660  CTTCTCACAGACGAACTTAGAGGAAAACGAAGCAGCAAGCAAGAAATTCGAACAAGCAGC  719
AtCGR3_NtCO  660  T..TAGC.....A...C.C..A..G..T.....C...TC.........T..G........  719

AtCGR3       720  TTCCAAGAGTTCATACAAACCAGCTTGTCAAGTTTTCCACCTCAAGCCATTACATTAG  777
AtCGR3_NtCO  720  CAGT...TCC..C.....G..T..A........G..T..TT.G.....T.....CGGT  777
```

Supplemental References

von Caemmerer, Susanne, and John R. Evans. 2015. "Temperature Responses of Mesophyll Conductance Differ Greatly between Species." *Plant, Cell & Environment* 38 (4): 629–37. https://doi.org/10.1111/pce.12449.

Ellsworth, Patrícia V., Patrick Z. Ellsworth, Nuria K. Koteyeva, and Asaph B. Cousins. 2018. "Cell Wall Properties in Oryza Sativa Influence Mesophyll CO2 Conductance." *New Phytologist* 219 (1): 66–76. https://doi.org/10.1111/nph.15173.

Evans, John R., and Susanne von Caemmerer. 2013. "Temperature Response of Carbon Isotope Discrimination and Mesophyll Conductance in Tobacco." *Plant, Cell & Environment* 36 (4): 745–56. https://doi.org/10.1111/j.1365-3040.2012.02591.x.

Nobel, Park S. 2009. "Chapter 8 - Leaves and Fluxes." In *Physicochemical and Environmental Plant Physiology (Fourth Edition)*, edited by Park S. Nobel, 364–437. San Diego: Academic Press. https://doi.org/10.1016/B978-0-12-374143-1.00008-9.

Xiong, Dongliang. 2023. "Leaf Anatomy Does Not Explain the Large Variability of Mesophyll Conductance across C3 Crop Species." *The Plant Journal* 113 (5): 1035–48. https://doi.org/10.1111/tpj.16098.
